# Phenotypical difference of Amyloid Precursor Protein (APP) V717L mutation in Japanese family

**DOI:** 10.1186/1471-2377-12-38

**Published:** 2012-06-15

**Authors:** Masao Abe, Naomi Sonobe, Ryuji Fukuhara, Yoko Mori, Shinichiro Ochi, Teruhisa Matsumoto, Takaaki Mori, Satoshi Tanimukai, Shu-ichi Ueno

**Affiliations:** 1 Department of Neuropsychiatry, Neuroscience, Ehime University Graduate School of Medicine, Shitsukawa, Toon-city, Ehime, 791-0295, Japan; 2 Department of Molecular Probes, Molecular Imaging Center, National Institute of Radiological Sciences, 4-9-1 Anagawa, Inage-ku, Chiba-city, Chiba, 263-8555, Japan

## Abstract

**Background:**

Alzheimer’s disease (AD) is the most common form of dementia. Mutations in genes such as those encoding amyloid precursor protein (APP), presenilin 1 and presenilin 2, are responsible for early-onset familial AD.

**Case presentation:**

In this study, we report a 275341 G > C (Val717Leu) mutation in the APP gene in a Japanese family with early onset AD by genetic screening. This mutation has previously been detected in European families. In the Japanese family we screened, the age at onset of AD was 47.1 ± 3.1 years old (n = 9; range, 42–52). The symptoms in the affected members included psychiatric vulnerability and focal signs such as pyramidal signs, epileptic seizures, and myoclonic discharges. An MR imaging study showed relatively mild atrophic changes in the bilateral hippocampus and cerebral cortices in all affected members compared with their clinical presentations.

**Conclusion:**

We conclude that the clinical features of Alzheimer’s disease can be different even when caused by the same mutation in the APP gene. Further clinical and genetic studies are required to clarify the relationship between phenotypes and genotypes.

## Background

Alzheimer’s disease (AD) is the most common type of dementia, with a prevalence of over 26 million worldwide [1]. According to the definition of National Institute of Neurological and Communicative Disorders and Stroke–Alzheimer’s Disease and related Disorders Association [2], AD is diagnosed based on detection of two of the following symptoms: amnesia, aphasia, apraxia and agnosia. Atrophic changes occur in the parietal and temporal cortices, especially the medial temporal lobe including hippocampus. The pathological changes in AD include amyloid plaques (include amyloid beta protein) and neurofibrillary tangles (including hyperphosphorylated tau protein).

Amyloid plaques consist primarily of amyloid β protein (Aβ), which is produced when APP is cleaved by β-secretase and then cleaved again by γ-secretase as part of the amyloidogenic pathway. According to the amyloid cascade hypothesis, an imbalance between Aβ production and clearance plays a role in the progression of AD. Aβ oligomers may directly inhibit hippocampal long-term potentiation and impair synaptic function in addition to the inflammatory and oxidative stress caused by aggregated and deposited Aβ as amyloid plaques. These processes lead to neurotransmitter deficits and the cognitive symptoms associated with disease progression [3,4].

The age of onset of AD is usually after the age of 65 years, but can be earlier if influenced by genetic mutations in familial Alzheimer genes. In early-onset familial AD, mutations in the genes related to the amyloid cascade, *amyloid precursor protein* (APP), *presenilin 1* (PS1), and *presenilin 2* (PS2), have been found. There are currently 32 known mutations in APP, 178 in PS1, and 14 in PS2, and these have been identified in 86, 392, and 23 families, respectively. Mutations in these three genes account for less than 1% of all AD cases [5]. Four different missense mutations (to an isoleucine, phenylalanine, glycine, or leucine) have been reported at codon 717, encoding valine, of the APP gene (for a review, see [6]). The Val717Leu mutation in APP has been reported in European-descendant families. In this study, we report the first Val717Leu APP mutation to be detected in a Japanese family, and describe the clinical signs and symptoms in detail.

## Case presentation

**Figure 1:**
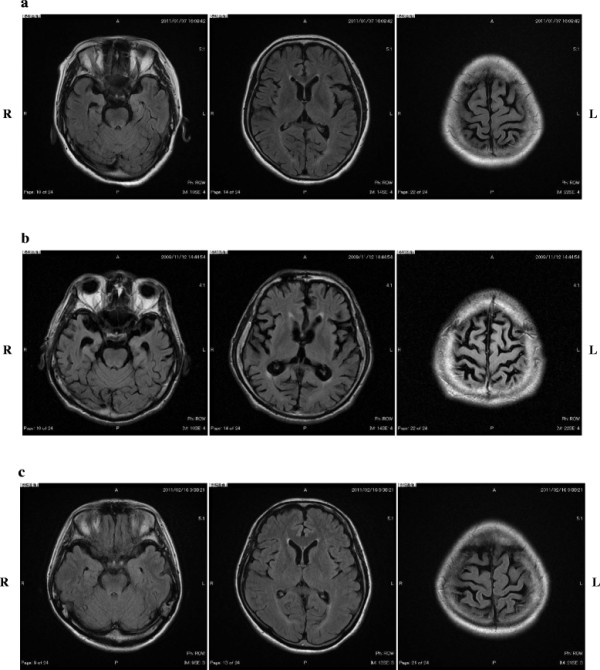
**Brain MRI (FLAIR). a) III-9 at the age of 55 years, b) the proband (III-10) at the age of 53 years, c) III-11 at the age of 52 years.** All siblings had generalized cerebral atrophy but this was mild relative to their clinical features.

**Figure 2:**
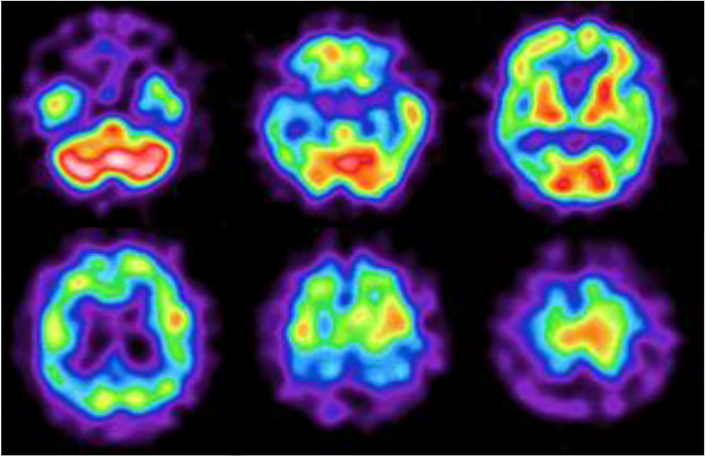
**I-IMP SPECT of the proband (III-10) at the age of 53.** A generalized decrease drop in cerebral blood flow is shown.

The proband developed deficits in short-term memory and loss of concentration at the age of 45. He was a banker with 16 years of education. When admitted to our hospital at the age of 47, the findings of a neurologic examination were normal and his mini-mental state examination (MMSE) score was 20/30. His Raven’s colored progressive matrices (RCPM) score was 30/36. Neuropsychological examination demonstrated intellectual decline with impaired memory, but preserved visuoperceptual skills. Brain MRI showed mild generalized cerebral atrophy. His conversation skills became gradually poorer and he had epileptic seizures several times in a year and developed gait disturbance at the age of 52. When he was 53 years old (six years after first admission), myoclonus and pyramidal signs were present. Neuropsychological testing could not be carried out at this point. Figures 1a and 2 show brain MRI and ^123^I-IMP SPECT findings, respectively.

## Family report

**Figure 3:**
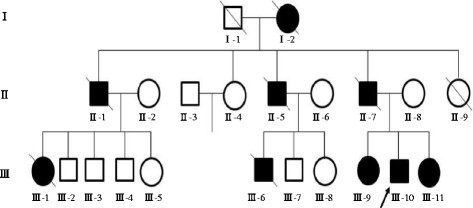
**Pedigree of a Japanese family with early-onset Alzheimer disease.** The arrow denotes the proband. The circles indicate females and squares indicate males. Solid symbols denote the affected individuals; open symbols denote unaffected individuals; slashes, deceased.

Figure 3 shows the 3-generation pedigree of a Japanese family with early-onset Alzheimer’s disease. The age at onset and death and unusual features are shown in Table 1. We gathered information from medical records of the participants including the proband (patient III-10).

**Table 1:** Ages of affected members

**Individual**	**Age at onset (years)**	**Age at death (years)**	**Other features**
I-2	50	65	not known
II-1	50	58	not known
II-5	42	56	not known
II-7	52	72	not known
III-1	47	54	attempt suicide
III-6	46	48	depression
III-9	45		disinhibition
III-10	45		seizure
III-11	47		poor insight
mean	47.1 ± 3.1	58.8 ± 8.5	

The proband’s elder sister (III-9) was a nurse with 14 years of education. She left her job at the age of 40 because she had trouble performing her work as a nurse. She showed clear short-term memory loss at the age of 45, which gradually worsened. At the age of 55, she began losing her way home and demonstrating agitation, aggression, irritability, lability, and urinary incontinence. She was admitted to our hospital for further examination. She angered easily, at anything, and needed to stay in a single room for special psychiatric care. Neurologic examination revealed pyramidal signs bilaterally and her MMSE score was 6/30. Brain MRI showed bilateral temporal and parietal atrophy (Figure 1b). She could not live by herself and was re-admitted to another hospital for long-term care.

The proband’s younger sister (III-11) was a housewife with 14 years of education. She was married and had a child. She presented to our hospital at the age of 52 years with a 5-year history of gradual progressive memory difficulties. Neurologic examination was normal without any psychiatric symptoms. However, her MMSE score was 24/30 and her RCPM score was 27/36. Brain MRI showed mild bilateral temporal and parietal atrophy (Figure 1c).

## Genetic analyses

Genomic DNA was extracted from peripheral blood leucocytes from five participants (II-8, a non-affected mother; III-7, a cousin; and III-9, 10, 11, the affected siblings) according to a standard protocol, after obtaining written informed consent approved by the Ehime University Ethics Committee. Written informed consent was obtained from the patient for publication of this case report and any accompanying images. A copy of the written consent is available for review by the Editor-in-Chief of this journal.

**Figure 4:**
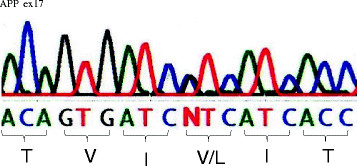
**Sequence of exon 17 of the APP gene in affected individuals.** The part of N shows that both G and C occur at nucleotide position 275,341 of the APP gene. This causes an amino acid change in codon 717 (valine to leucine).

**Figure 5:**
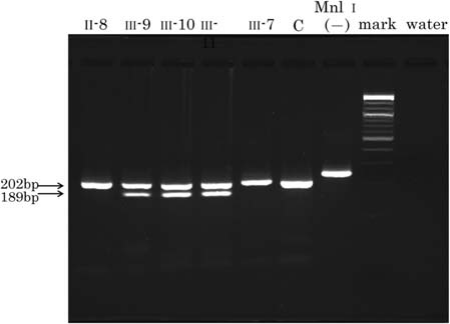
**The results of 4% agarose gel electrophoresis of Mnl I-digested PCR products.** The mutant product is cut into two fragments of 202 and 189 base pairs (bp) by Mnl I digestion after PCR. Individuals III-10 (proband), III-9 and III-11 carry the mutant allele. C represents a normal control subject.

At first, it was planned to analyze the mutations of known genes causing AD in the proband. Based on the report of [Finckh et al. 7], direct sequencing of the *PS1* exons 1 to 12 and *APP* exons 16 and 17 was done because those mutations account for more than half of family-based autosomal-dominant Alzheimer’s dementia. The resulting sequences were compared with those in normal control subjects. Sequencing analysis of the proband (III-10) revealed no mutations in the PS1 gene. The following primers were used for amplification and sequencing of exon 17 of the APP gene: forward, 5′ ccaaatgtcccctgcatt 3′ and reverse, 5′ gaaacatgcagtcaagtttacct 3′. The PCR master mix was composed of 12.5 μl reactions containing 0.5 U of AmpliTaq Gold (Applied Biosystems, Austin, TX), 1.25 μl of 10× PCR Buffer, 2.5 nmol of dNTP mixture, 2.5 nmol of each primer and 100 ng of genomic DNA. The conditions for amplification were as follows: an initial denaturing step of 94°C for 10 min, followed by 45 cycles of 94°C for 30 s, 57°C for 30 s, 72°C for 1 min, and a final elongation step of 72°C for 9.5 min. The sequence of exon 17 of the APP gene revealed a single nucleotide substitution in one allele: 275341 G > C (amino acid: Val717Leu) (Figure 4). The resulting 189-base pair product was assessed by PCR-restriction fragment length polymorphism (PCR-RFLP) analysis using *Mnl*I and run on a 4% NuSieve 3:1 agarose (Takara Bio, Japan) (Figure 5). This mutation was not observed in 50 normal control subjects (100 normal chromosomes). 

## Conclusions

By screening exon 17 of APP and the coding exons of PS1 and PS2, a mutation detection rate of ~57% [7] can be achieved in patients with suspected familial AD (FAD), a figure comparable to those of earlier reports (46–71%) [8-10]. The family described here was had a V717L mutation in exon 17 of APP. This is the first finding of this mutation, which has been reported in several European descendent families [3,11], in an Asian family. In the family, age at onset of AD was 47.1 ± 3.1 years (mean ± S.D.; range, 42–52, n = 9) and age at death was 58.8 ± 8.5 years (mean ± S.D.; range, 48–72, n = 6). These findings are different from the data reported by [Murrell et al. 3], in which age at onset was ~38 years (range, 35–39) and age at death was ~46 years (range, 40–50), but it is similar to data reported by [Godbolt et al. 11], in which age at onset and death were ~50 (range, 48–57) and ~61 (range, 57–68), respectively. Disease duration was the same, being ~10 years in all three families. Because ApoE status was shown to affect the age at onset or death and clinical features in the precedent studies, the ApoE status of the family members was analyzed. The ApoE genotypes in III-9, III-10 and III-11 were ϵx3/ϵx3, ϵx3/ϵx3 and ϵx3/ϵ x3, respectively [12]. Thus, it seemed that the ApoE status was not responsible for the features in this family. In this Japanese family, the first symptom was progressive short-term memory impairment, as observed in typical AD. However, there were focal signs and symptoms that were unusual for AD; for example, frontal lobe impairment (depression, apathy, disinhibition) in III-6, III-9 and III-10, pyramidal signs in III-9 and III-10, and epileptic seizures and myoclonus in III-10, although there were no focal lesions in either brain MRI or SPECT. Godbolt *et al.* reported that atypical features, seizures and hallucinations were apparent in several patients with the APP Val717Leu mutation [11]. We suggest that this mutation affects pathways other than the amyloidogenic pathway to modify the features of AD, and that some genes and/or environments may change the features of AD, even with the same APP mutation.

Further clinical and genetic studies are required to clarify the relationship between phenotypes and genotypes and to identify additional biological factors, such as tau, that may have a role in the pathogenesis of AD.

## Competing interests

The authors declare that they have no competing interests.

## Authors’ contributions

MA, YM, SO carried out the molecular genetic studies, participated in the sequence alignment. NS is a treating neurologist of the patient, and made a contribution to acquisition and interpretation of data. MA, NS have been involved in drafting the manuscript, while RF, TM, TM, ST revised it critically for important intellectual content. SU supervised this study, participated in its design and coordination, and revised the manuscript that led to the final approval of the current submission. All authors read and approved the final manuscript.

## Pre-publication history

The pre-publication history for this paper can be accessed here:

http://www.biomedcentral.com/1471-2377/12/38/prepub
